# Monitoring and Fault Location Sensor Network for Underground Distribution Lines

**DOI:** 10.3390/s19030576

**Published:** 2019-01-30

**Authors:** Antonio Parejo, Enrique Personal, Diego Francisco Larios, Juan Ignacio Guerrero, Antonio García, Carlos León

**Affiliations:** Department of Electronic Technology, University of Seville, 41011 Seville, Spain; epersonal@us.es (E.P.); dlarios@us.es (D.F.L.); juaguealo@us.es (J.I.G.); antgar@us.es (A.G.); cleon@us.es (C.L.)

**Keywords:** sensor network, smart grid, power line monitoring, underground distribution lines

## Abstract

One of the fundamental tasks of electric distribution utilities is guaranteeing a continuous supply of electricity to their customers. The primary distribution network is a critical part of these facilities because a fault in it could affect thousands of customers. However, the complexity of this network has been increased with the irruption of distributed generation, typical in a Smart Grid and which has significantly complicated some of the analyses, making it impossible to apply traditional techniques. This problem is intensified in underground lines where access is limited. As a possible solution, this paper proposes to make a deployment of a distributed sensor network along the power lines. This network proposes taking advantage of its distributed character to support new approaches of these analyses. In this sense, this paper describes the aquiculture of the proposed network (adapted to the power grid) based on nodes that use power line communication and energy harvesting techniques. In this sense, it also describes the implementation of a real prototype that has been used in some experiments to validate this technological adaptation. Additionally, beyond a simple use for monitoring, this paper also proposes the use of this approach to solve two typical distribution system operator problems, such as: fault location and failure forecasting in power cables.

## 1. Introduction

In the last few years, electric systems have become one of the backbones of the modern world. In order to afford this complex scenario, the paradigm of Smart Grids [1,2] was proposed, adding a new hierarchical organization to make its management possible. A general description of today’s conventional electric delivery system can be broken down into mostly isolated components of generation, transmission, substation, distribution, and the customer [3]. Focusing on distribution networks, it is the part of power system that connects primary substations to costumers, and which is divided into two levels; the primary distribution network (at Medium-Voltage, MV) and the secondary distribution network (at Low-Voltage, LV). Specifically, the primary distribution network is responsible for connecting primary substations (from High-Voltage to Medium-Voltage, HV/MV, conversion) to secondary substations (from Medium -Voltage to Low-Voltage, MV/LV, conversion).

Taking this structure into account, a blackout in one feeder of this top level of this distribution network would affect to a high number of consumers, causing inconveniences to them and the utility company. A proof of its importance is the existence of some indicators whose mission is to assess the quality of Distribution System Operator (DSO) service, e.g., System Average Interruption Frequency Index (SAIFI) and System Average Interruption Duration Index (SAIDI) [4]. Some governments have established minimum legal requirements about supply quality, and these requirements can be mainly described using the two mentioned indicators, or other similar ones (e.g., [5]). Due to their importance, it is essential for primary distribution lines to be sensorized and monitored allowing the utility companies to find problems and solve them as soon as possible. This strategy complies with one of the main objectives of a Smart Grid [6] (improve its power quality).

In this sense, it will be necessary to increase the grid infrastructure to provide new information to the Distribution Management System (DMS) of the DSO. Thus, this new monitoring system should cover maintenance in two ways: reactive and predictive:

(A) From the point of view of reactive maintenance, it must be able to detect and locate possible faults and incidences in power systems. This information is essential for the Outage Management System (OMS) in order to minimize their impact on the customers.

While fault detection requires specific disconnection systems (for the quick line disconnection in order to minimize damages in lines) and are traditionally deployed in substations, fault location is a broad field of study and there are multiple examples of methods that can be applied in every situation. In [7], the author explains how fault location methods can be applied over Smart Grids, while in [8], a review of different fault location methods and their behavior is shown when they are specifically applied on underground distribution systems. 

All these techniques follow the paradigm of self-healing, this is, the grid itself automatically rapidly senses, detects, analyses, responds and then restores the power supply [9]. This philosophy is also known as Fault Location, Isolation and Service Restoration (FLISR) [10]. Unfortunately, most of these fault location methods are typically not applicable because they usually lack information or have problems in scenarios with distributed generation (with bidirectional power flows). In this sense, as it will be detailed in the next sections, this paper proposes taking advantage of a new approach based on a proposed new distributed instrumentation which use its distributed character to solve the fault location problem.

(B) From the point of view of predictive maintenance, it must be able to monitor the aging and degradation of supply systems, allowing DSOs to identify critical sections that could presumably suffer future problems. In [11], it is shows how work and environmental conditions play an important role in the successful prediction of the lifespan of the cable line. The correlation between ambient factors and aging of the cable insulation has been presented in some other papers. In [12], Du et al. performed a study of how temperature affects to tree growth in epoxy resin insulation, from their apparition to breakdown stages. Park also studied epoxy and other materials to correlate their breakdown strength with temperature [13], showing how higher temperatures reduce breakdown strength. In [14], Kruizinga et al. analyzed polymeric insulation degradation due to humidity and other chemical factors. Kemari et al. proposed a methodology for the evaluation of XLPE and PVC/B insulations using harmonic analysis and expose a correlation between aging and temperature [15]. In this sense, this paper proposes taking advantages of the proposed instrumentation, which uses its distributed character, and the inclusion of new measurement parameters to improve the fault forecasting. 

Obviously, from both perspectives (reactive and predictive approaches), a monitoring system is needed. The idea of deploying a complementary metering infrastructure to solve this type of task is not new. One of the most widespread examples of its use can be seen over the secondary distribution network, where an Advanced Metering Infrastructures (AMI) with smart meters has been deployed to gather customer power consumption automatically. There exist multiple papers in this field, as [16], in which the authors propose the use of a Wireless Sensor Network (WSN) in the electrical energy-measurement structure. In [17], Lloret et al. propose an integrated Internet of Things (IoT) architecture for smart meter networks to be deployed in Smart Cities. Referring to the power transmission system, reference [18] shows how wireless sensor technology can be used to assess the mechanical health of transmission lines.

However, this type of monitoring is restricted to certain aspects, such as power measurement, and it is unable to commit the rest of descripted operations to help keeping the electric system working. The lack of these could be solved using a sensor network, which is composed of a large number of sensor nodes, which are densely deployed either inside the phenomenon or very close to it [19]. Sensor networks have a lot of applications [19], one of them being ubiquitous and pervasive monitoring of an environment. The application of sensor network technologies is an emerging alternative with numerous contributions in the last few years, both in general [20,21] and specific ambits, such as overhead line monitoring [22], distributed generation system monitoring [23] and electric vehicle charging management [24].

Specifically, for distribution lines and Smart Grid monitoring, there are multiple examples of the application of sensor networks. Reference [25] explained the necessity of implementing Wide-Area Monitoring, Protection and Control (WAMPAC) and why these systems will become indispensable in the future of power systems. Reference [21] reviewed the application of WSNs for electric power systems and studies the statistical characterization of wireless channel in different power system environment, such as substations or power control rooms. In [26], Pinto et al. proposed a WSN deployment to improve electrical secondary substation using ZigBee technology and integrating a communication model based on IEC 61850.

As can be seen, these types of monitoring are already available mainly for substations and overhead distribution lines, but not for underground distribution lines. In the first environment, the sensor devices are more accessible, being typically located outdoors, so many papers propose using WSN. Its application over underground lines would require using external antennas, so it complicates the deployment of the network using this technology.

However, there are also authors that expose ideas for the monitoring of underground distribution lines. In [27], the authors propose a method to evaluate cable aging by locating incipient faults in underground distribution cables. However, these types of approaches require fast and expensive instruments, so they are not adequate for deployment over distribution lines because of the high number of measurement devices required by them. Other authors propose the application of diverse methods of fault location over underground primary distribution lines. As an example, [28,29] expose methods for that, but they require voltage and current measurement. 

To solve this problem, this paper proposes the deployment of a sensor network over underground primary distribution power lines. This kind of deployment allows the DSO to monitor the three-phase currents of each underground power feeder, but also the inclusion of more types of sensors is considered. In this sense, this paper proposes a sensor network architecture which is adapted to distribution grid specification. Moreover, this work also proposes a sensor prototype and raises several experiments to validate its operation in this field. In addition, it also raises as an example for the proposed sensor approach, its use for solving the two DSO problems, such as: fault location and cable failures forecasting.

In summary, this paper is organized as follows: Section 2 analyzes the characteristics of underground primary distribution lines. In Section 3, the sensor network architecture and its justification are presented. Section 4 explains the sensor node structure and how it works internally. The synchronization system applied in the sensor network is shown in Section 5. Section 6 describes the constructed sensor prototype. Section 7 describes the fault location problem and proposes a fault location algorithm and its advantages, as a use case of this sensor network. Then, Section 8 details the experiments showing the behavior of the sensor network. Lastly, Section 9 concludes this paper.

## 2. Underground Primary Distribution Lines Structure and Characteristics

The first step to understand the proposed architecture of nodes is to briefly study the characteristics and limitations associated with the environment where they must be deployed. Specifically, the type of cables that make up underground lines, the way they laid out and their structure. In this sense, a deployment of sensor nodes over underground primary distribution lines is more difficult than overhead lines due to their characteristics.

**Figure 1:**
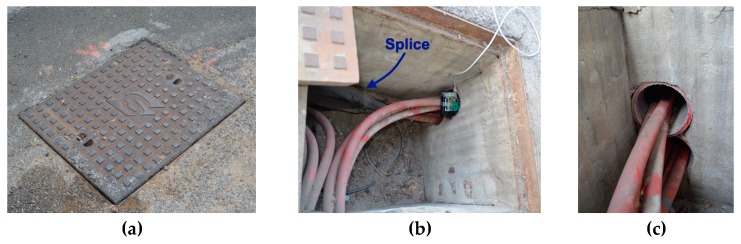
(**a**) Manhole cover; (**b**) manhole for a line direction change; and (**c**) cable layout in the underground duct (view from the manhole).

Firstly, unlike overhead lines whose topology allows them to be uncovered, underground lines as the name suggests has its layout buried in the ground. Thus, for reasons of maintenance and future extensions, these facilities should be carried out under certain conditions. Among these stands out the recommendation to install manholes every certain distance or at every direction change (especially in urban environments). An example of this recommendation can be found in [30], where the U.S. Department of Defense (DoD) establishes a limit of 120 m. In other cases, as in Spain, the utilities restrict this maximum distance between two manholes to 40 m. It is interesting to note that this distance determines (in a breakdown case), the cable segment or section to be replaced, because the splices should be made at these manholes. An example of the appearance of a box and its interior layout can be seen in Figure 1. In any case, this type of structure does not allow using wireless communication methods, except mounting additional systems out of manholes, which could be complicated in cities.

Secondly, due to their isolation needs, distribution lines require an insulating cover using cables instead of wires. Thus, for security and durability, they cannot be punctured at any point, restricting the points where it is possible to make stress measurements at the points where, for construction reasons, the conductors are accessible (e.g., secondary substations). This fact limits the application of method that use direct voltage measurement (i.e., impedance-based methods) at these points, unlike the overhead facilities where these measures are easily accessible everywhere along the line. These cables contain a core, an insulator, a sheath or protective sheath and a jacket. One of the most usual mountings, especially for short lines, is to connect the two extremes of the sheath to the electrical earth. Thus, it forms a Faraday cage, inhibiting the use of electrostatic methods to measure voltages. This restriction limits the improvement in the accuracy that these methods could experience when considering their application along the line in a distributed manner. 

**Figure 2:**
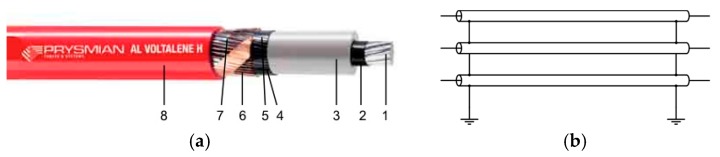
(**a**) Underground wire (AL RHZ1-OL, Prysmian™). (1) Aluminum conductor, (2) Intern semiconductor, (3) Insulator, (4) Extern semiconductor, (5) Water protection, (6) Cupper sheath, (7) Separator, (8) External cover; and (**b**) most common sheath connection for short segments of underground MV primary distribution lines in Spain.

An example of this type of cable is RHZ1-OL (Figure 2a), used by Endesa [31,32] (one of the most important utility companies in Spain) for their underground primary distribution facilities. There exist multiple recommended ways of connecting cable sheaths to the earth, as can be observed in [33]. They can be connected through a sheath voltage limiter that is only connected when the voltage is over certain values, or they can be connected directly to the earth at the same point, which is known as a solid connection or a single-point bonding (there is a common point connecting the three sheaths). When a solid connection is applied in both ends of the cable, it is called multiple single-point bonding. It can be done with or without cross-bonding [34].

One of the most common mounting for short segments of underground medium voltage lines (e.g., urban lines) is the solid connection of the sheath to the earth through two pikes, which are located at both ends of the cables at secondary substations, without applying cross-bonding, as shown in Figure 2b.

In a structure of this type, thinking about the deployment of a network of sensor nodes, to solve the communication between nodes along the lines, it is convenient to choose an option that does not require an additional complex infrastructure to work, because of the characteristics of the primary distribution network, so Power Line Communication (PLC) appears to be a good option. The PLC technology is being widely used in Smart Grids ambits (typically using capacitive coupling) because it takes advantage of the existing wiring to carry out the communication. In this sense, some authors propose taking advantage of this technology beyond a simple communication. In [35], Huo et al. proposes a solution to monitor and detect cable degradations using PLC and machine learning techniques. In [36], Ercan et al. discussed a solution to send PLC data through distribution transformers. They use the transmission line model (TLM) technique to study the high frequency behavior of the transformer. In [37], González-Sotres et al. analyzed the performance of PLC-PRIME protocol for AMI purposes over low voltage networks. 

## 3. Sensor Network Architecture

As described above, this work proposes the design of the distributed sensor network technology, adapting this technology to solve some problems of the power distribution grid. In this sense, the most basic application is the network monitoring. Obviously, for this purpose, the network elements must be able to measure electrical network parameters. However, due to cable restrictions (as discussed in Section 2) the voltage measurement is discarded, so that current measurements will only be made. Additionally, and taking advantage of the distributed approach of the sensor network, more types of sensors have been proposed. Following the approaches reviewed in Section 1, humidity and temperature could be useful parameters to determine the aging of the wiring, so its use seems interesting for failure forecasting. Moreover, other problems that can take advantage of this distributed approach are the fault location methods.

In any case, to perform these analyses, the nodes must be able to establish a network and communicate with each other. To make this communication, assuming the existence of the cable, PLC was raised as an alternative. Notwithstanding, PLC are usually injected using voltage signals, mainly over low-voltage lines, where voltages are directly accessible. In primary distribution lines their voltages are not accessible due to their danger, so this communication mode cannot be applied.

Instead, the option of injecting current communication signal by means of inductive methods is available. As was shown before, the major part of short primary distribution lines in several countries (e.g., in Spain) have both extremes of the cable sheath directly connected to the earth, creating a closed path with a low impedance. This loop offers a closed path through which a current injected by an inductive coupling can circulate and using this to establish the communication between nodes. Taking this into account, the proposed method to communicate network nodes is inductive PLC. Another problem that it must be considered is the measurement method of currents. Inductive method has been chosen as the most appropriate measurement technique, having into account that it only requires an inductive coupler around every cable to perform measurements. In this way, the wire will not be manipulated and security normative will be respected.

**Figure 3:**
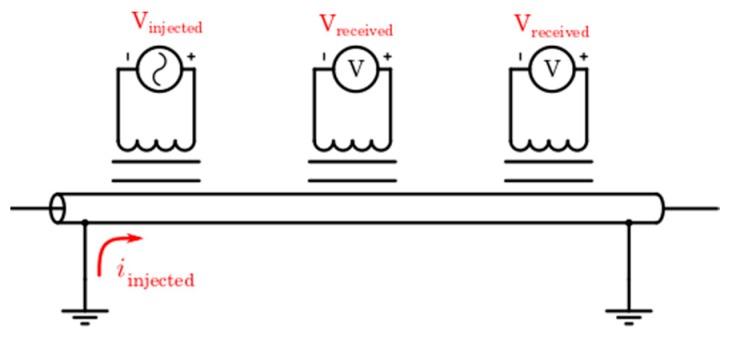
PLC through the sheath and the earth in primary distribution line cables.

As can be seen in Figure 3, the connection of cable extremes to the earth creates a closed path with low impedance. This path can be exploited by a PLC system, using an inductive coupling (with no direct contact). This architecture allows a communication between the nodes in the same segment line (between secondary substations). Thus, this communication is restricted mainly to its loop (the communication path with lowest impedance). Besides, due to the existence of a Global Earthing System (GES) [38], it is possible that communication affects the next sections. However, this influence decreases with the distance (because the path impedance increases with the loop length). Thus, the interference between sections only affects the nearby sections.

Thanks to its low impedance, a low voltage signal is enough to inject the current communication signal in the circuit. In fact, the proposed nodes use this communication method with voltage signals in the range of 3.3 V.

Moreover, there must exist linker nodes near each secondary substation to connect the different segments of the feeder and one linker node more in the header (or end) of it, acting as Base Station to connect to the DSO information systems. Both linker nodes are basically normal nodes with an additional communication port to make the connection possible.

**Figure 4:**
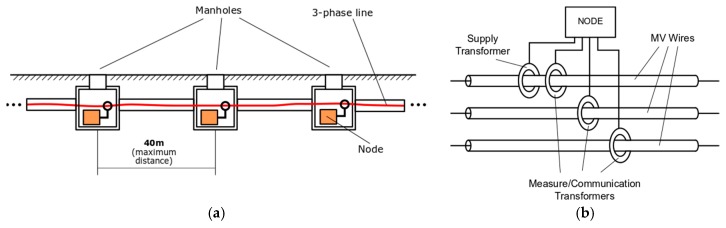
(**a**) Primary distribution line segment; and (**b**) Node installation in a manhole.

Therefore, an initial basic schema of the proposed network is as shown in Figure 4. A node is initially installed in every manhole along the line. To install a node, a current transformer is coupled around each cable and another one (supply) is coupled only to one of them (does not matter which one is used).

Optionally, this infrastructure can be reduced, installing a smaller number of sensors. This action reduces the economic investment. However, as will be described in the next sections, it will also have effect on accuracy of the proposed analyses.

Thus, it is interesting to highlight the design of a current transformer that allows measurement and communication simultaneously. In the case of the measurement transducer, a sensor is required for each cable. Contrary, for communication, only a single cable would be necessary (obviously common for all nodes). However, a short-circuit or cut in the cables (typical in a power fault condition) make the communication difficult. Due to this, and assuming the need of a measurement element per cable, the proposed nodes use three couplers and can automatically select one of these to communicate. This topology makes the communication channel redundant and simplifies the installation and configuration process. Thus, the combination of measurement and communication using the same current transformer is not trivial and will be explained in detail in Section 4.1.

Additionally, another discussion is related to the need to use an additional current transformer as a power supply adapter instead of using also the measurement and communication transformers for this task. However, the energy harvesting process modifies the transformer flux dramatically, so that unlike the communication process, it is difficult to measure and supply simultaneously with the same core. However, in a fault location study the useful information lasts only a few network cycles (typically between 3 and 5 cycles) so that the measurement process must be continuous. Thus, both uses cannot be alternated and require additional elements for energy extraction.

**Figure 5:**
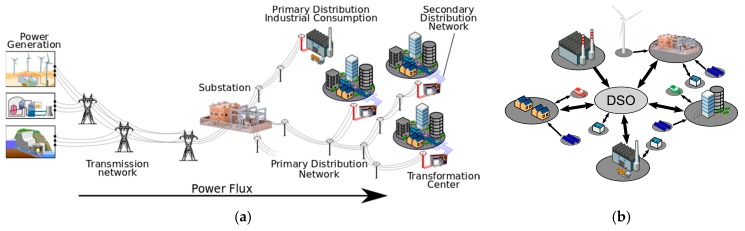
(**a**) Traditional distribution line topology; and (**b**) Smart grid topology.

Every node can measure currents in the line in both directions, it does not matter if the power flows from utility to customers or vice versa. Therefore, another advantage of this system is that it can be installed in both, over traditional distribution systems with one-direction energy flows (Figure 5a) and over Smart Grid topologies (Figure 5b), where energy flows can take multiple directions. 

Despite the objective of these nodes is fault location, they can also be used to get temperature and humidity data of cables. This data can be used to detect high-temperature problems, excessive humidity in manholes (which could be due to floods), etc. When there is a large amount of data available, they can also be used to analyze cable aging related to temperature and humidity.

Thus, the usefulness of the node does not end here. It should be understood as a tool to expand the applications to be carried out. Proof of this idea is the optional lighting sensor that has also been added, thinking about an application to detect the manhole manipulation.

**Figure 6:**
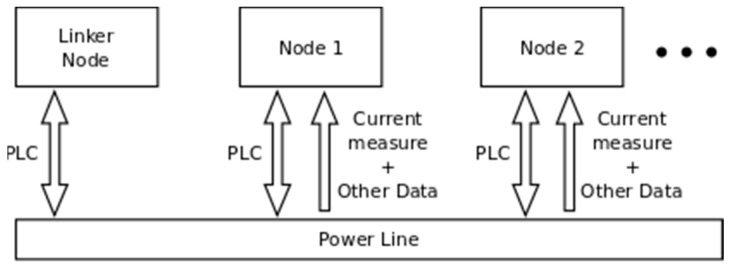
Proposed sensor network architecture.

In Figure 6 the structure of information exchange is depicted. Every node performs measurements (currents and other type of variables, such as temperature or humidity) and communicates with the others to execute a collaborative task (e.g., fault location). Moreover, there is at least one linker node for every line segment (base station or intersegment connector at secondary substation), which shares the information amongst the different nodes along the power feeder. Additionally, the base station node acts as a gateway between the network and the outside to allow extraction of information. The main information that should be extracted is fault location alarms.

This section and the previous ones have shown the characteristics of primary distribution lines and the characteristics that sensor nodes must have. The next section exposes how nodes are designed to accomplish all the described requirements and functions.

## 4. Sensor Node

The previous sections expose the sensor network, the environment where it must be deployed and its associated problems. It also explains all the general functions that the nodes must perform and how they affect each other. Considering these indications, this section shows the internal structure of a sensor node and how every part works.

**Figure 7:**
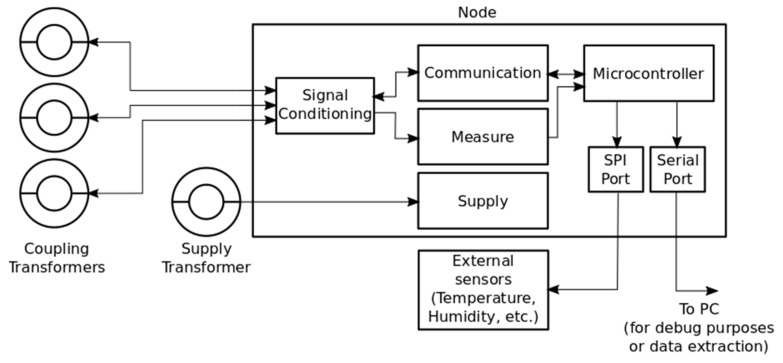
Internal architecture of sensor node.

A sensor node is composed by a few subsystems. This fact can be observed in Figure 7.

As can be seen, some parts are interconnected to share energy or information. Information interfaces are the coupling transformer, which connects the node with the power line, and the serial port, which allows the connection of other devices such as a personal computer to give orders to the nodes and extract information. A supply transformer performs energy harvesting from power lines to supply energy to the node, using a battery to keep its energy level. This is important to maintain the node connected while there is a blackout (typical after a fault event), which makes it impossible to extract energy from lines.

### 4.1. Coupling Transformer

Usually, in this type of application it would be necessary to use independent cores for communication and measurement. Using the same core for both functions is not an easy task, due to impedance differences for every task.

On the one hand, the measurement of current requires very low equivalent impedance on the secondary winding. Otherwise, the high primary current would cause very high voltage in this winding. On the other hand, PLC requires very high equivalent impedance on secondary winding, in order to allow that all the injected energy goes through the communication path (primary side), not through the secondary. Nevertheless, even in an only-communication device, power currents would cause very high voltages over the secondary, as was mentioned before.

As can be seen, both functions have contrary requirements of impedances. In this way, some authors have proposed methods to allow inductive PLC solving the problem of the low frequency currents. In [39], Murata and Kimura proposed a method to avoid core saturation. It consists of separating the two parts of the core with a gap to eliminate low frequency flux transmission through the core. Only very high frequency, which is used to communicate, can be transmitted to the core. The problem of this solution is that the cores can only communicate, not allowing simultaneously measurement of currents.

**Figure 8:**
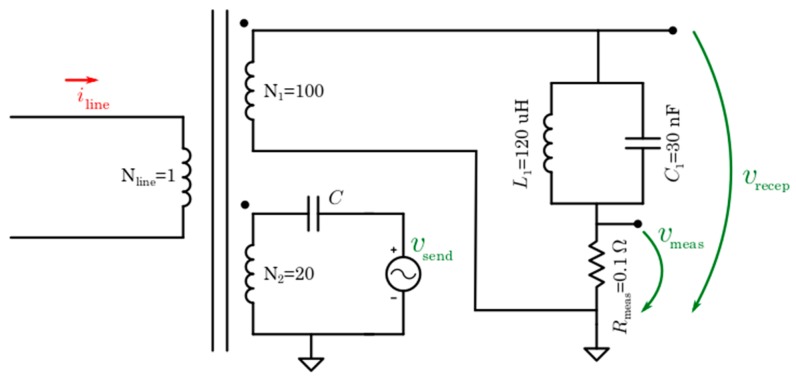
Communication-measurement connection.

One of the advantages of the developed device is that it can perform measurements and communication simultaneously over the same transformer. A solution that allows both functions working simultaneously can be done thanks to a resonant filter on the secondary coil of the ferrite core. This idea is shown in Figure 8.

Resonant LC filters present a peak of very high impedance (theoretically infinite) at their resonance frequency and lower impedance at the other frequencies. This frequency depends on inductance and capacitance values according to equation:

(1) $$\omega_{0}=\frac{1}{\sqrt{L\cdot C}},$$

where the width of the impedance peak depends on the quality factor of the filter (named *Q*). It is equal to:

(2) $$Q=\frac{R}{2\cdot \pi \cdot f\cdot L}=2\cdot \pi \cdot f\cdot C\cdot R=R\cdot \sqrt{\frac{C}{L}}$$

Finally, the impedance value is equal to:

(3) $$\left|Z\left(\omega \right)\right|=\frac{\omega }{C\cdot \left(\omega^{2}-\omega_{o}{}^{2}\right)},$$

The PLC modem uses Spread Frequency-Shift Keying (S-FSK), which means it uses frequency modulation to transmit information. Specifically, it uses two frequencies: the space frequency fs (data 0), and the mark frequency fm (data 1). In this case, it is configured to use frequencies of 85.5 and 86.5 kHz, so the filter must have the impedance peak at 86 kHz (in this mode, both frequencies are into the peak). Moreover, it needs a quality factor that allows a very low impedance for 50 or 60 Hz frequencies (frequency of power lines).

**Figure 9:**
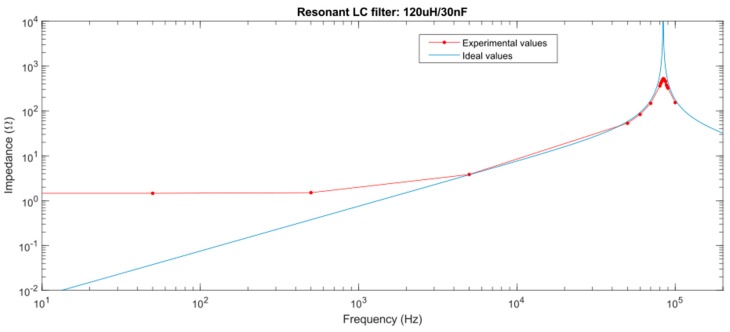
Resonant filter impedance values.

Selected values are 120 µH of inductance and 30 nF of capacitance. The filter has the impedance profile shown Figure 9. In this, the ideal values model the impedance of an ideal LC filter (without considering any resistive component), therefore its impedance at 86 kHz tends to infinite (due to the parallel resonance). The experimental values show differences due to the internal resistances of the chosen coil and capacitor. An approximation of the real values is a coil of 120 µH with a series resistance of 1.5 ohms (affecting low frequency band in the figure) and a capacitor of 30 nF and a parallel resistance of 800 ohms (limiting the parallel resonance). Considering these values, the experimental results are very similar to the described situation.

Thus, the real filter present adequate impedance values, this is, low impedance at 50 Hz and high impedance at 86 kHz.

Multiple experiments were performed using different core materials and form (ferrite and soft iron). The chosen has been a rounded ferrite core due to its low losses, linearity and good behavior both in low and high frequencies.

**Figure 10:**
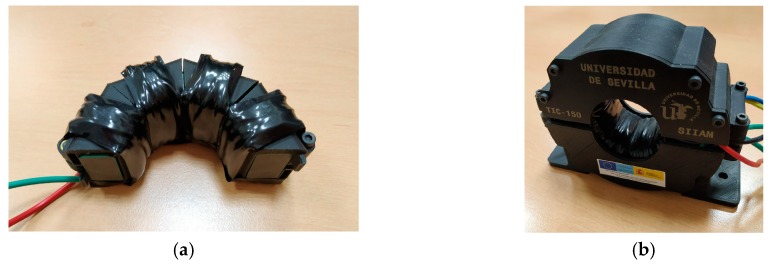
(**a**) Half ferrite core and its coils; and (**b**) whole protected coupler.

Using this core, the two coils (of 100 and 20 turns) are coupled using ABS pieces, as shown in Figure 10a. The whole set is protected by a protective ABS box, as can be observed in Figure 10b.

This coupler is completely adapted to be easily mounted around primary distribution cables and connected to the node. As it has been explained, this resonant filter structure affects the current measurement and communication subsystems. Their characteristics are clarified in the next sections.

### 4.2. Current Measurement Subsystem

A current transformer is used to measure current though every wire. These current transformers divide by 100 the main current and it is measured by a shunt resistor.

While measuring current, whose frequency is 50 Hz (typical in Europe), the impedance of resonant filter is very low, much lower than the shunt resistor, and so it does not interfere with the current measurement. This happens because the resonant filter is adjusted to show a high impedance peak at the frequency of 86 kHz. In this way, the peak covers frequencies of 85.5 and 86.5 kHz, which are the frequencies used for communication.

### 4.3. Communication Subsystem

The core of the communication system is an AMIS49587 modem (ON Semiconductor, Phoenix, AZ, USA). It is a Spread-Frequency Shift Keying (S-FSK) PLC modem. The main problem of PLC technology is the generation of electromagnetic noise. Due to this, the European Committee for Electrotechnical Standardization (CENELEC) has defined in standard EN50065 [40] a series of bands in electromagnetic spectrum where the use of PLC is allowed. These bands are described in Table 1.

This modem uses a synchronization method based on zero crossings of the line signal (50 Hz in this case). This information is used to establish communication between different devices. 

As was explained before, communication frequency will be 85.5 and 86.5 kHz, so these frequencies are located into band A (reserved for energy utilities). The amplitude of communication signals is about 1 V of peak.

Moreover, this modem can apply encryption to messages and perform CRC checking over received messages. These functions give security and robustness to the network.

### 4.4. Energy Supply Subsystem

All the nodes of a sensor network must have a source to provide enough energy to work. In this case, the source could be one of the three primary distribution power cables. 

As explained before, these cables are covered by a protective jacket that cannot be punctured due to the fact that it would degrade its durability dramatically. Thus, every sensor node is connected to a supply transformer that is coupled to one of the three power cables, allowing the energy extraction through an inductive coupling. As previously mentioned, this transformer is specific for energy harvesting purposes, and it does not realize any other function than that. It is connected to a rectifier bridge, to obtain DC voltage, and lastly it is connected to the bq25570. It is totally independent from measurement-communication transformers.

Obviously, this transformer can only obtain energy while there is current through power lines, so during a blackout (i.e., after a fault event) the node will not have additional energy supply. To solve this problem, every node includes a Li-ion battery which can be charged when there is enough available energy, or discharged when required, having a capacity of 2600 mAh. 

The energy extraction capacity of the system depends mainly on the type of coupler used. In this paper, the chosen one has been a ferrite core (same size that current sensor couplers) with 250 copper turns. This configuration has been considered enough, but slight changes in the coupler would make it possible to extract more power.

As will be shown later, the system can charge the battery even with 25 A (around 5 or 10% of the nominal current). However, this current value could be lower in extreme cases, such as the end of the lines. In these zones, it could be necessary to change the coupler configuration. Furthermore, in these cases, it is also possible to modify the node consumption policy (restricting its communication to the minimum). In any case, this system requires a minimum current, otherwise it would not be applicable, or it would require external power. I.e. extracting the energy from the secondary substation (which restrict its deployment to the line segment ends).

To study this behavior, an analysis of the battery charge under different situations with 25 A has been done in Section 8.3.

### 4.5. Microcontroller Subsystem

The microcontroller subsystem manages the whole described functions of the node. It can manage communication, perform continuous current measurement and communicate through a serial port (for debugging purposes or data transferring).

The core of this subsystem is a STM32F100RB microcontroller, manufactured by STMicroelectronics (Geneva, Switzerland). It is based on Cortex-M3 architecture.

The combination of lower energy consumption and a good performance makes it adequate for this system. It includes all necessary peripherals (multiple channel ADC, timers and ports) and count with several low consumption modes. In these modes, a microcontroller can reduce its consumption to very low levels. This is useful when the micro is not performing any task.

### 4.6. External Measurement Subsystem

This subsystem contains other sensors used to measure environment variables, such as temperature and humidity. Thanks to the SPI bus, the microcontroller can read and process these two parameters and allows the node to management addition sensors, if any application requires other variables in the future.

## 5. Synchronization System

In a sensor network, a fundamental requirement is a method to establish time synchronization between nodes. In the proposed case, synchronization is used to allow comparison between current measurements made by different nodes. The reason why synchronization is needed is that power lines transport sinusoidal currents, so measurements must indicate signal phases considering a common reference. This means that signals must be referred to the same time base.

Moreover, the PLC modem AMIS49587 uses a type of synchronization referred to the 50 Hz (or 60 Hz) electric signal of the line. Taking into account that this device is used to locate faults, it must continue communicating when there is no electrical signal in lines.

In normal conditions, this modem was conceived to operate over low voltage lines (typically with capacitive coupling). As can be appreciated in the datasheet, it has an input that allows the detection of zero crossings in electrical signal. Using these zero crossings, the modem establishes its PLC synchronization with other modems of the network. In the case of a blackout, this synchronization is not possible, implying communication loss.

In the proposed case, the modem must always have a signal to detect zero crossings and not to lose communication. This is especially problematic during the occurrence of a blackout, when the nodes must communicate the information about the last measurements to locate the fault.

To solve this problem, we have discarded a direct connection between the electrical signal and the modem. Instead of it, a signal follower has been installed to connect them.

Following this idea, the signal follower will be constantly converting the electrical main sine signal of 50 Hz to a square signal of the same frequency and phase. If a blackout or any other anomaly in the signal occurs, the signal follower detects it and will maintain a 50 Hz frequency from the last valid cycle of signal. This square signal guarantees that the modem will have a reference signal to establish synchronization with other modems, at least during a certain time after the blackout occurs. This time depends on two long modems can maintain their synchronization before their divergence make their time gap too much long. This time is about one minute after the blackout. Notwithstanding, even when the synchronization is lost, some techniques can be used to reestablish communication, e.g., change clock phase of a modem to reduce clock gaps.

**Figure 11:**
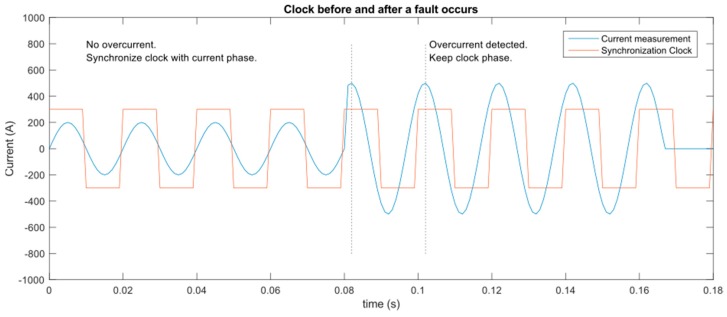
Clock synchronization using current measurements.

Figure 11 shows how clocks are synchronized with current phases while there is no overcurrent detected. When an abnormal behavior is detected in the current measurement, the clock is fixed to 50 Hz without changing their phase (currents’ phases are measured, but not used for clock synchronization purposes). It allows modems to keep their clocks synchronized during some time after the fault.

About the comparison among current measurements, it is done referring to their phases to the synchronization clock.

## 6. Prototypes

The described nodes have been constructed and tested. Additionally, a graphical user interface (GUI) was created. 

The prototypes have the capacity of monitoring three lines at the same time, and it can harvest energy from one of these lines.

**Figure 12:**
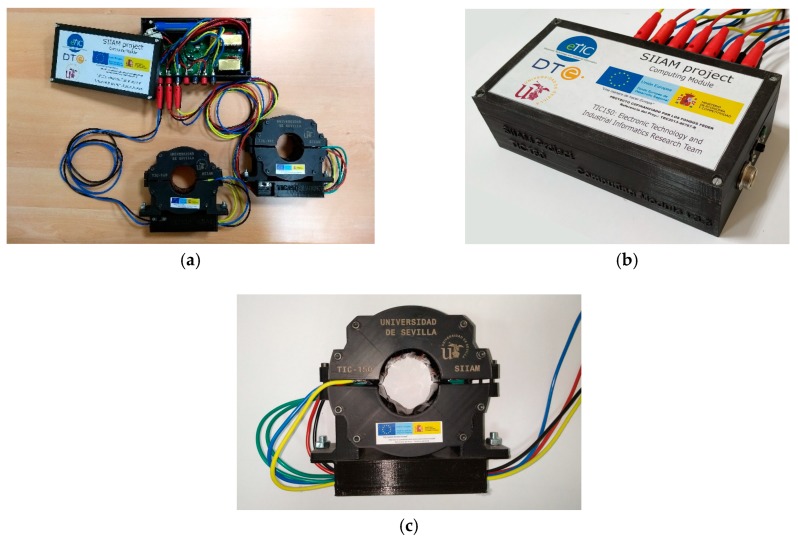
Node sensor prototype: (**a**) Node (opened); (**b**) Node (closed); and (**c**) Coupler.

All the circuits are included in a single Printed Circuit Board (PCB) and protected in a box. Figure 12a shows an opened node (internal circuit are visible) with only two transformers connected. Figure 12b shows a closed node with all the four couplers connected.

The monetary cost of this prototype and its elements is related in the Table 2.

### Computer Interface

**Figure 13:**
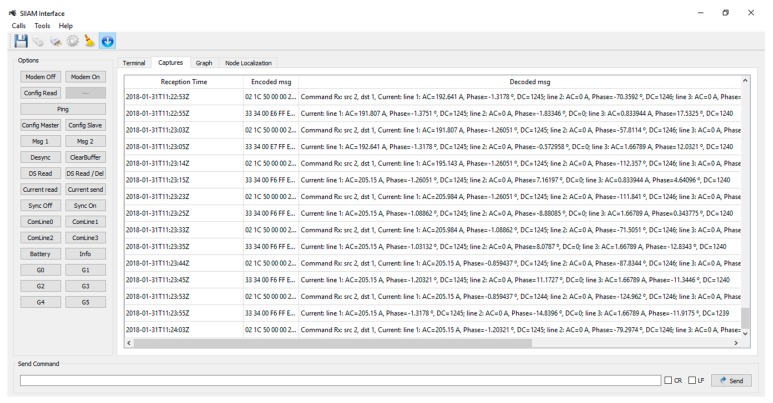
Computer interface. Frame captures.

A computer interface allows a more comfortable use of the nodes. This interface (Figure 13) has multiple buttons to execute different orders and operations over the nodes (e.g., get information, send messages over PLC, etc.). It also has multiple tabs to watch raw frames (Terminal Tab), decoded frames (Captures Tab) and information about current measures (Graph Tab). This application allows ones to export the complete set of data from the session on CSV format. In this way, the obtained information could be analyzed later.

## 7. Fault Location Methods and Distributed Sensor Network Approach

As was noted above, fault location techniques are a strategic priority for the DSOs, due to an improvement in them causes a reduction in the fault isolation and service restoration times. These techniques have been classified into three families: based on traveling waves [41], frequency components analysis [42] or impedance-based methods [8].

Traditionally, the first two families of these methods are usually applied in transmission feeders because they require expensive devices and complex analyses to locate the fault position which is incompatible with distribution topologies (more numerous and more ramified than the level of transmission). The third family is based on impedance measurement during the fault and the estimation of the fault distance based on the cables model. This family of methods requires a cheaper instrumentation and are typically applied in distribution grid. 

**Figure 14:**
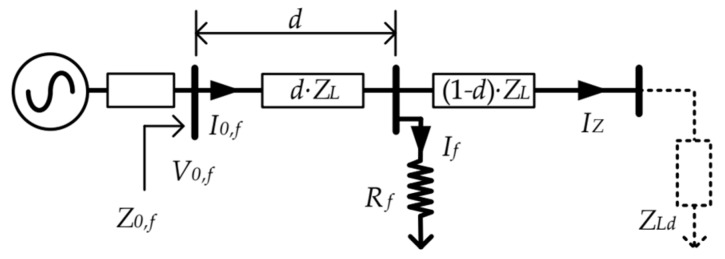
Basic operating principle of impedance-based location method [8].

In summary, impedance-based (or apparent impedance) methods assume that the fault contact has a purely resistive behavior. Thanks to this, the estimation of the fault position is focused on the analysis of the imaginary part of the measures. Figure 14 and Equation (5) shows this procedure, one of its most basic and easier approach (using the reactive component method [43]).

(4) $$V_{0,f}=d\cdot Z_{L}\cdot I_{0,f}+R_{f}\cdot I_{f};\;\mathrm{where}\;I_{f}\gg I_{Z}=>I_{0,f}≅I_{f}$$

(5) $$d≅\frac{Im\left[Z_{0,f}\right]}{Im\left[Z_{L}\right]}=\frac{Im\left[V_{0,f}/I_{0,f}\right]}{Im\left[Z_{L}\right]}$$

However, they have the other inconveniences such as multiple solutions (possible points with the same impedance along different branches) and low accuracy (due to its high sensitivity to measurement and line modeling errors) problems [8]. Additionally, to these problems are added the difficulties that they have when distributed generation (essential in a smart grid) is introduced. These generation spots replace the traditional unidirectional flow with new bidirectional flow profiles which demand a greater number of measurements and complicate even more their interpretation. 

As an alternative, this paper proposes taking advantage of the distributed sensor network approach, using numerous low-cost sensors along the lines to solve the problem. This approach aims to highlight a clear advantage of using the sensor network proposed in this paper.

### 7.1. Location Algorithm of Each Node

**Figure 15:**
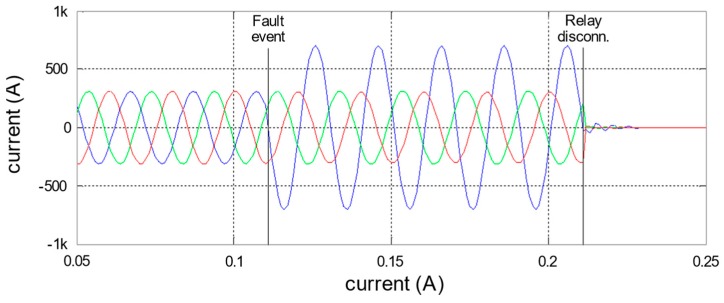
Example of a fault stages registered by a sensor node.

The location algorithm is based on a current balance between electrically neighbor nodes during the fault situation. Specifically, each sensor node is continually measuring until a fault event is detected. Thus, the line disconnection requires a fast response. Therefore, this action is directly done by the protection relay, using itself detection system. Figure 15 shows an example of this node registration under a fault situation. In these signals, it is possible to clearly identify two moments: when the fault happens and when the protection relays disconnect at the headers.

**Figure 16:**
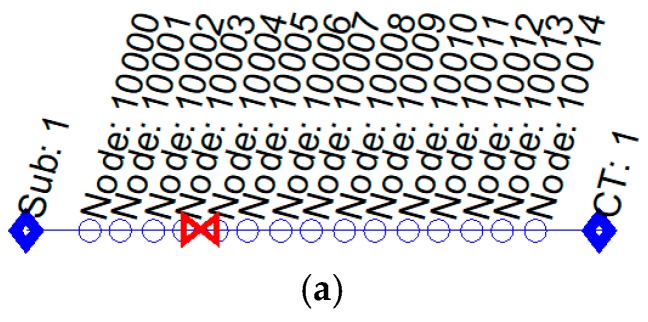

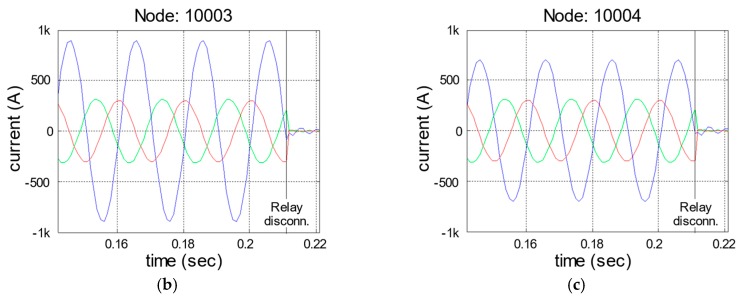
Example of a Fault situation: (**a**) Nodes’ topology; (**b**) data registered by nodes 10003 (at left of the fault position); and (**c**) data registered by nodes 10004 (at right of the fault position).

As described above, each node is continually analyzing the current measurements. When some of them detect a fault pattern (a sudden increase in the measured current, maintaining it during some cycles and with a subsequent disconnection), the node considers the detection of a fault. Later, after the relay disconnection, only the sensor nodes, which have detected the fault start a communication process. In this dialogue, each node communicates its current measurement to its electric neighbors and they answer with their measurements. An example of these signals recorded by the nodes before and after the fault position is shown in Figure 16. The Figure 16a represents a feeder segment that connects two secondary substations and in which a simple fault (line-ground) happened between nodes 10003 and 10004. Figure 16b shows the data registered by Node 10003 (at one side of the fault position), and Figure 16c shows the data registered by Node 10004 (at the other side of the fault position).

It is interesting to note that, the measurement of all nodes at the same side of the fault have similar values. Therefore, the nodes that identify any difference between its measurement and the value communicated by one of its neighbors will be identified as ends of the affected segments.

In addition, another conclusion that can be extracted from the registered data is that is not necessary to transmit the complete signal waveform, just exchanging the module or RMS value of the current is enough to determine the searched nodes. This fact is another advantage of this approach since it greatly relaxes the synchronization constraints between the nodes to compare their values in a consistent manner. It allows the nodes to avoid the need of using specific and expensive hardware for this purpose (i.e., the Global Positioning System, GPS, devices in Phasor Measurement Units, PMUs). Moreover, the deliberate delay added to the communication during the fault event minimizes the effects that this phenomenon could have on the communication process, assuming a GES.

### 7.2. Accurancy of the Location

As previously commented, regardless of the problems associated with the distributed generation, the most extended location methods in the distribution grid are the impedance-based family. However, these methods are very sensitive to inaccuracies in the cables modeling, and due to their methodology, they are extremely sensitive to numerical errors, associated with the treatment of low impedances values (typically low in cable models), getting errors around of 10%, but it is often greater in branched networks where it is even more difficult to estimate the corrected line segment [8].

Conversely, the alternative proposed in this paper uses other location approaches. It has the advantage of being independent from the cable characterization and does not have the numerical errors previously mentioned. Specifically, the accuracy in the location is directly defined by the number of deployed sensors installed. Being able to choose this according to the needs of the application the best tradeoff between location accuracy and deployment cost. 

In the same way, this approach has an additional advantage since it increases the location robustness. Specifically, a failure in one node does not incur a loss of the detection capacity. This failure only incurs a reduction on the location accuracy because, when it does not respond, its neighbor looks for their next nearest electrically neighbor nodes. Therefore, it only extends the line segments length included between the new association.

Thus, another important issue to be considered is the influence of the current measurement error and how it may have effects on the identification of the line segment under fault (e.g., an error in the measurement of one of the sensors could be associated to a current imbalance resulting from a fault). However, this fact is not a real problem. The initial situation without any fault (or without any real current imbalances) allows the sensor network to identify these errors previously, and autocalibrates them for a subsequent proper operation.

## 8. Experimental Results

Using the two constructed nodes, it has been possible to perform some experiments to prove the correct behavior of these nodes.

### 8.1. Communication Over an Earth Connected Wire

As was described before, the communication channel of this sensor network is the sheath of medium voltage wires, which is connected in its two sides to the earth (theoretically, a perfect conductor).

**Figure 17:**
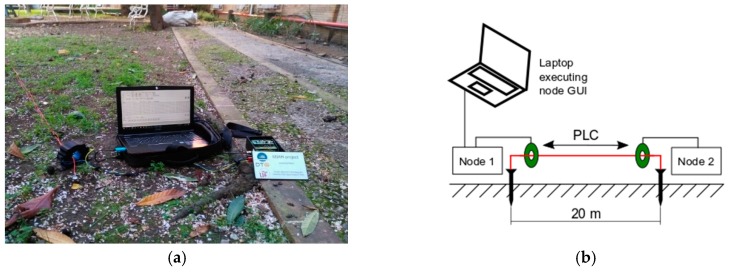
(**a**) Coupled node and laptop; and (**b**) experimental setup.

This first experiment (Figure 17) had the objective of this test, if it was possible to use this channel in PLC. A simple wire of 20 meters was connected to the earth using two small pikes (a quarter part of a standard pike) and two nodes (single-phase nodes) were coupled to the wire.

The result of this experiment shows that communications could be realized without problems in both directions. As expected, our test demonstrates that the earth is a low impedance path for PLC communications, even using small ad out of norm pikes. In real scenarios, the earth path must be even better.

Moreover, additional experiments including different impedances values into the path indicates that this type of communication works without any problem for impedances until 580 ohms. This resistance value is higher than the standardized maximum value of earthing impedance and the sheath impedance of the cable. Therefore, assuming the worst case and the impedance per meter of the cable sheath, the PLC system can work in line segment (communication loop) of up to 2 km. Obviously, this is a system limitation for networks with long line segments. However, this distance is typically enough for urban environments where the distance between secondary substations is usually less than 1 km. 

### 8.2. Measurement and Communication

To locate faults, it is essential to compare measured currents between pairs of electrical neighboring nodes. It implies measuring the current continuously and communicate it every certain time.

**Figure 18:**
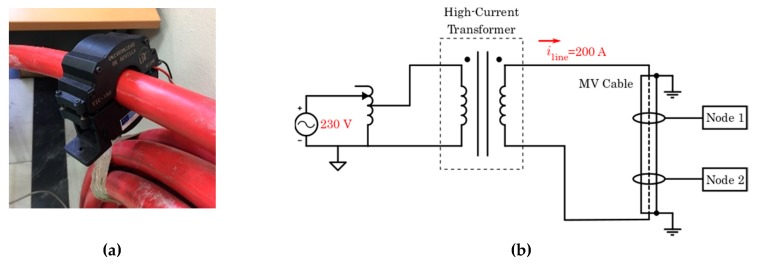
(**a**) Coupled transformer; and (**b**) high-current transformer.

In this experiment (Figure 18) a piece of standard underground primary distribution (MV cable) cable (XLPE of 150 mm^2^) connected to a high-current transformer was used. This connection allows one to inject up to 250 amperes through the cable. Two nodes were installed over the standard cable, using the energy harvesting transformer and a measure-communication coupler.

One of these nodes sent its measures every 10 s over PLC. The other node sent these measures and its own measures to a PC. The value of current was changed along the experiment (from 50 A to 200 A).

**Figure 19:**
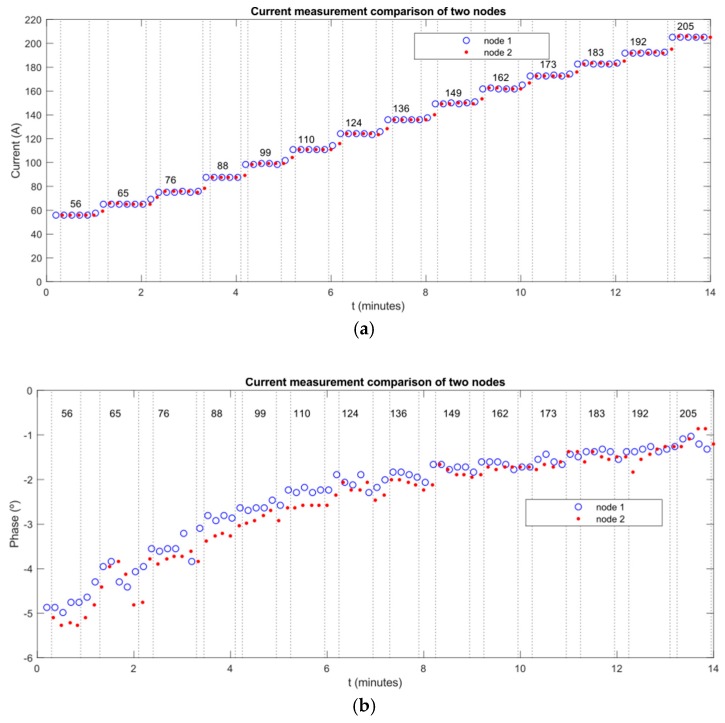
(**a**) Amplitude graph; and (**b**) phase graph.

In Figure 19a,b we can observe the values of amplitude and phase are obtained. In both cases, the obtained measures of both nodes were very similar. This implies that there is no any problem (fault) in the line between these two nodes.

**Figure 20:**
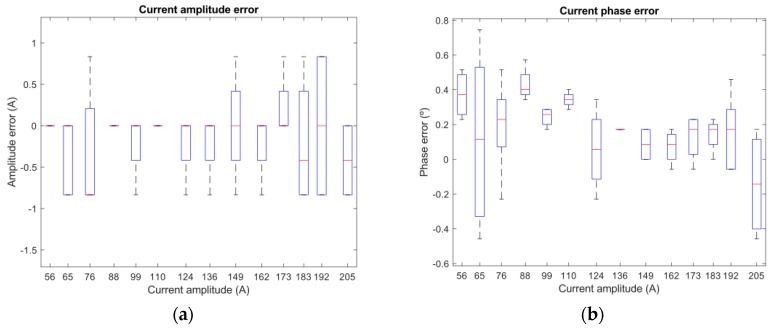
(**a**) Amplitude error; and (**b**) phase error.

Figure 20a,b show deviations of amplitude/phase differences between measurements of two nodes. They are separated for every different value of current from 56 A to 205 A. Maximum obtained deviations were 0.834 amperes (for amplitudes) and 0.745 degrees (for phases).

The results of this experiment indicate that the synchronization between nodes is strong enough to let us compare two signals (amplitude and phase measures) and locate faults in lines using the proposed location algorithm.

### 8.3. Energy Harvesting

The energy harvesting subsystem must provide enough power to keep the nodes alive during a normal function of the lines. Moreover, it needs a battery to operate during a certain amount of time after a fault. The sensor node contains a Li-ion battery that has a capacity of 2600 mAh. 

As described below, the system has been designed to be able to charge the battery during its normal functioning period when the current through lines is 25 A (but it can be designed using a bigger core to charge the battery even with lower current). Taking this into account, we can calculate the maximum number of PLC messages that could be sent, and the maximum reception period that can be used.

**Figure 21:**
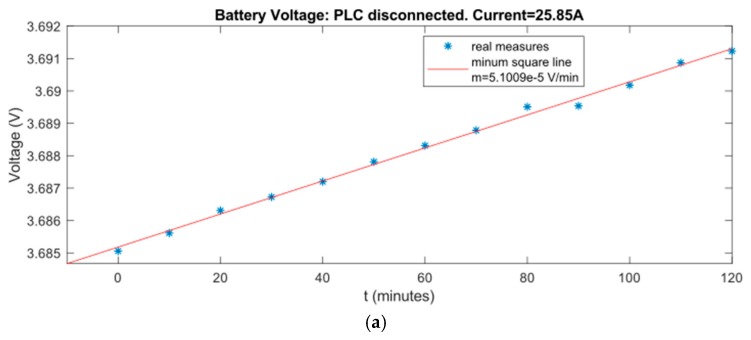

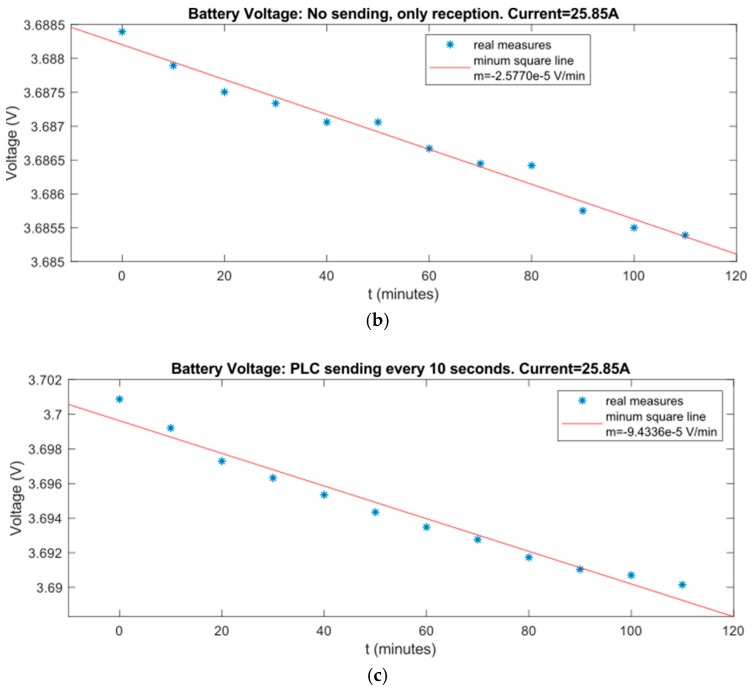
(**a**) Battery voltage: PLC disconnected; (**b**) Battery voltage: only reception; and (**c**) Battery voltage: sending and receiving.

The battery of a node has been monitored during every type of situation to watch the its charge speed during these conditions. This data can be used to estimate charge speed. The results can be seen in Figure 21.

Moreover, node consumption has been measured in multiple use cases. Table 3 contains consumption results while there is not current through power line, so the node is unable to extract more energy. Table 4 contains data while the node is harvesting energy (25 A through power line). Both consider three situations: modem OFF (no PLC communications), modem ON (the node can receive, but it does not send anything) and PLC sending (modem is ON and it is sending messages). 

Using this set of data, it is possible to determine how much energy is required for every functionality of the node and configure the time in which every mode can be used.

Using this data, it is possible to estimate how much time a node can be receiving, or sending messages while the battery maintains its State of Charge (SoC). Considering that in a minute the node only activates PLC reception (modem ON) during a certain time (t_on_), and PLC are OFF the rest of the time (t_off_), the maximum time is the result of equation:

t_on_ · I_on_ = t_off_ · I_off_, (6)

>

t_on_ · 15 = (60−t_on_) · 6, (7)

t_on_ = 17.14 s, (8)

If in a minute the node activates PLC sending during a time (t_send_), and it powers off the modem the rest of the time (t_off_), this maximum sending time (where every message last approximately 1.2 s) can be known using the following equation:

t_send_ · I_send_ = t_off_ · I_off_, (9)

t_send_ · 52 = (60−t_off_) · 6, (10)

t_send_ = 6.2 s, (11)

and the maximum number of messages (n_mes_) that can be sent would be equal to:

n_mes_ = t_send_ / t_mes_ = 6.2/1.2 = 5.16, (12)

so, the maximum number of messages that can be sent by the same node during a minute (without losing battery charge considering 25 A in lines) is 5 messages.

This number of messages is enough for the nodes to maintain clock synchronization over the line. In this way, when a problem occurs, all the nodes will be able to communicate again and locate the fault.

The last use case that must be considered is the maximum time a node can keep itself running during a blackout. The battery capacity (with 2600 mAh) was conveniently selected to allow a very long time of operation, even without obtaining more energy. Considering that the battery is fully charged (it will be, while the nodes respect all the described restrictions), the maximum time of operation after a blackout, considering the maximum consumption possible (sending messages continuously) is equal to:

t_max_ = C_battery_ / I_send_ = 2600 mAh / 70 mA = 37 h, (13)

so, the maximum time is 37 hours. This is more time than necessary to perform fault location operations.

## 9. Conclusions

There exist multiple possibilities for the monitoring of overhead medium voltage lines, but there is not so much in the field of underground lines. The main difficult is the type of cable used, which cannot be punctured at every point, so the voltage measurement is not allowed in these cases.

To solve this problem, this paper proposes a distributed sensor network based on several nodes deployed over underground primary distribution lines, especially useful in urban environments. This sensor network can continuously monitor the power lines and measures currents in a non-invasive way. This information is directly aligned with the Smart Grid philosophy and has a direct application for fault location (reducing the time response to a failure) and for cable aging studies by means of temperature and humidity data (allowing a predictive maintenance).

From a technological point of view, this paper demonstrates, using a real prototype, that the adaptation of distributed sensor network technology to a primary underground distribution grids is possible. In this sense, the communication between nodes has been solved using PLC by a new measurement-communication structure. This structure allows both functions work at the same time using only one coupler per line. Its key is a resonant filter that presents high impedance at PLC frequency to allow PLC sending/reception and very low impedance at power frequency that allow current measurements. It is interesting to note that typical approaches of other authors do not execute these two functions over the same couplers, this approach being one of the main contributions of this work. This achievement required the design and construction of current transformers specifically designed for this research. The selected material for the core has been ferrite due to its better behavior and lower losses than in soft iron cores. 

The proposed sensor node has been designed to be completely autonomous and can be easily installed by inductive couplers in manholes. In this way, experimental results show that this system could be completely autonomous even with relatively low power currents and can effectively communicate the obtained information through primary distribution lines without puncturing.

Finally, as a proof of the usefulness of this new instrumentation, two possible applications have been proposed as use cases. The first one is a fault location method that takes advantage of the distributed character of the proposed network to solve this problem. The second one is focused on the failure forecasting in power cables by means of the local information of multiple variables to model their aging, such as: humidity and temperature.

## Figures and Tables

**Table 1:** CENELEC bands.

Band	Frequency (kHz)	Use	Access Protocol
A	3–95	Reserved for energy suppliers.	No
B	95–125	Indoors use.	No
C	125–140	Reserved for domestic networks.	CSMA/CD
D	140–148.5	For alarms and security systems.	No

**Table 2:** Prototype cost.

Part	Price (€)
Node	53.05
Current Sensor (x3)	17
Power coupler	6
Temperature sensor	1.3
Humidity sensor	2.5
Light sensor	1.8
TOTAL	79.85

**Table 3:** Node consumption while not harvesting energy.

State	Consumption (mA)	Battery Duration (h)
Modem OFF	12	217
Modem ON	33	78
PLC sending	70	37

**Table 4:** Node consumptions while harvesting energy (current of 25 A through coupled power line).

State	Consumption (mA)	Battery Duration (h)
Modem OFF	−6	infinite ^1^
Modem ON	15	173
PLC sending	52	50

^1^ Battery in charge.
